# Acute myeloid leukaemia at an early age: Reviewing the interaction between pesticide exposure and *KMT2A*-rearrangement

**DOI:** 10.3332/ecancer.2017.782

**Published:** 2017-11-30

**Authors:** Maria S Pombo-de-Oliveira, Francianne Gomes Andrade, Gisele Dallapicola Brisson, Filipe Vicente dos Santos Bueno, Ingrid Sardou Cezar, Elda Pereira Noronha

**Affiliations:** Pediatric Hematology-Oncology Program, Research Center, Instituto Nacional de Câncer (INCA), Rio de Janeiro 20231-050, Brazil

**Keywords:** acute myeloid leukaemia, KMT2A-rearrangements, pesticides maternal exposure, gene polymorphisms

## Abstract

Acute myeloid leukaemia (AML) in early childhood is characterised by a high frequency of recurrent genomic aberrations associated with distinct myeloid subtypes, clinical outcomes and pathogenesis. Genomic instability is the first step of pathogenic mechanism in early childhood AML. A sum of adverse events is necessary to the development of infant AML (i-AML), which includes latency of biochemical-molecular and cellular effects. Inherited genetic susceptibility associated with exposures to biotransformation substances can modulate the risk of DNA damage and it is a very important piece in the pathogenic puzzle. In this review, we have aimed to explore the chain of events in the time-points of the natural history of i-AML, which includes maternal exposures during pregnancy, the speculations about the formation of somatic mutations during foetal life and the secondary genomic aberrations associated with i-AML. The modulation of risk conferred by xenobiotic metabolism´s genes variants is the bottom line of the pathogenic process. Since we have conducted observational and molecular investigations in early childhood leukaemia, the data focused here is based on Brazilian findings with summarised results of our experience with epidemiological and molecular studies in early-age leukaemia.

## The profile of genomic aberrations found in early-age AML

Acute myeloid leukaemia (AML) accounts for about 20% of childhood leukaemia. There are increased incidence rates occurring in infants (less than 1–2 years of age, i-AML). The incidence declines dramatically in children between five and nine years of age at diagnosis, and then it increases again in adolescents and young adults [1].

AML is a heterogeneous disease that develops through the transformation of a haematopoietic progenitor cell as a result of a block in differentiation and uncontrolled proliferation, leading to an accumulation of immature cells and the decrease of mature blood cells. It was postulated that AML might be originated by mutations in genes involved in proliferative and signal transduction pathways, as well as transcription factors of progenitor myeloid cells [2, 3]. The major AML subtypes are originated from chromosomal rearrangements with fusion genes, as elegantly described by Balgobind *et al* [3]. In settings such as infant AML (i-AML), there are strong evidences that aberrant fusion genes initiate during foetal life, although the majority of the biological investigations were demonstrated in acute lymphoblastic leukaemia (ALL) samples [4].

The most frequent chromosomal abnormalities in children with AML (c-AML) older than two years are those associated with the core-binding factor (CBF) aberrations, such as t(8;21)(q22;q22)/*RUNX1-RUNX1T1* and inv(16)(p13.1;q22)/*CBFβ-MYH11*. Acute promyelocytic leukaemia (APL) with t(15;17)(q22;q21)/*PML-RARA* is also frequent in children [5]. In recent analysis of i-AML series in Brazil, the *RUNX1-RUNX1T1*, *CBFβ-MYH1* and* KMT2A* (or* MLL*) rearrangements (*KMT2A*-r) and *PML-RARA* occurred in frequencies of 12.2%, 17.4%, 67.6% and 3.2%, respectively [6]. Amongst infants, CBF-group and APL are rare, and the most common morphological subtypes are myelomonocytic (AML-M4/M5) and megakaryocytic leukaemia (AML-M7). The recurrent *KMT2A-*r is highly prevalent in our series of i-AML cases. Other chromosomal translocations, such as the t(7;12)(q36;p13)/*MNX1*-*ETV6*, t(8;16)(p11;p13)/*MYST3-CREBBP*, t(1;22)(p13;q13)/*RBM15*-*MKL1* and inv(16)(p13.3q24.3)/*CBFA2T3*-*GLIS2*, are all specifically associated with i-AML in variable prevalent frequencies that encompass the remaining 30% of aberrations [7–11].

The great majority of i-AML clinically presents with high white blood cell counts, hepatosplenomegaly, often central nervous system involvement and chloroma. A subset of i-AML has pre-leukaemia or constitutional syndromes that suggest heterogeneous etiologies for these cases. Indeed, approximately 10% of i-AML has myelodysplastic syndrome with monosomy 7 or del(7q), has Down Syndrome (DS), has Noonan syndrome or carry neurofibromatosis type 1 mutations, which all predispose to early AML [12, 13]. Children with DS have an increased risk of developing leukaemia, and among neonates and infants, AML is associated with *GATA-1* recurrent mutations [14–16]. *GATA-1* gene encodes a transcription factor and the mutation results in a truncated form of the protein GATA-1 that in physiological haematopoiesis interacts with other myeloid-lineage regulator genes. *GATA-1*mutations associated with trisomy 21 are crucial events to initiate megakaryoblastic abnormal proliferation [14, 15]. Evidence shows that likewise *KMT2A-*r, the *GATA-1* mutation occurs during foetal life [17]. *GATA-1* mutation is present in transient abnormal myelopoiesis (TAM), a clonal pre-leukaemia condition that occurs in about 10% of neonates with DS, presenting at a median age of 3–7 days with the accumulation of megakaryoblasts, increased leukocytes and thrombocytopenia. The majority of TAM regresses spontaneously without treatment; however, about 20% of these patients will develop megakaryocytic leukaemia in the first four years of life [18, 19]. This group of patients is often excluded in clinical and epidemiological studies that explore risk factors associated with etiopathogenesis of AML.

Since we have performed clinical, epidemiological and molecular studies in early-age childhood leukaemia, this review focuses on the development and data from maternal exposures during pregnancy and molecular events associated with i-AML (children with less than 24 months, excluding AML associated with Down Syndrome).

## Therapy-related myeloid leukaemia as proxy for maternal exposures during pregnancy associated with early molecular events in infant myeloid leukaemia

Given the young age of most children with i-AML, it was proposed and it is consensually accepted that early-age leukaemia is initiated before the birth of the child. The main evidence for a prenatal origin of childhood leukaemia was described in identical twins with concordant leukaemia. The same genomic fusion gene sequences were identified in twin pairs with acute leukaemia, which led to the assumption of a common clonal origin. The plausible basis for this finding is that in one twin foetus, clonal progeny spread to the co-twin via the vascular net. This assumption was endorsed by the identification of clonotypic gene fusion sequences, such as *KTM2A-AFF1* and* ETV6-RUNX1*, in dried neonatal blood spots of children who subsequently developed leukaemia [20, 21]. It is clear that acquired genetic abnormalities, especially those gene fusions are the most frequently found genetic alterations in early infancy (< 24 months of age). It is unlikely, however, that a single chromosome translocation or gene mutation itself would be enough to cause overt leukaemia, and alterations in other classes of genes that impair cell differentiations (RAS-MAPkinase) probably cooperate with the survival of a malignant clone. Deregulation of the MAPK genes signalling caused by somatic mutations and particularly *RAS* mutation was associated with i-AML and chemical exposures [2, 3].

Based on biologic similarities between the therapy-related myeloid neoplasm (t-MN) and infant leukaemia with similar *KMT2A-r* caused by topoisomerase II (Topo-II) inhibitors [22], the t-MN leukemogenesis could be proxy to modelling studies associated with biochemical-molecular events in the early stage of i-AML with *KMT2A-r*. In t-MN investigations, the dose-effect associations can be well documented with qualitative and quantitative measurements, while in i-AML, the associations’ estimates are a presumption of biological plausibility.

Patients with a t-MN have clonal genomic abnormalities in their bone marrow cells, which frequently are correlated with the type of preceding cytotoxic compound, the amount of drug exposed and the latency period. The classical t-MNs occurred after exposure to alkylating agents, Topo-II inhibitors and/or radiation therapy, and were described after a latency period of 5–10 years, often preceded by a myelodysplastic phase [22]. Nowadays, several reports on t-MN have unraveled the multistep process, suggesting that drugs interfering with DNA remodelling by Topo-II inhibitors can mediate the formation of specific chromosomal translocation breakpoints. Chemotherapy agents targeting Topo-II enzymes increase the steady-state levels of cleavage complexes by preventing religation of the transient Topo-II break, eventually leading to apoptosis and cell death. It increases the chances of DNA lesions being repaired by nonhomologous end joining, leading to chromosomal translocations (mainly the 11q23 region). Chemotherapy used to treat primary malignancies, in particular, Topo-II inhibitors, leading to specific gene mutations, creates a genetic frame on which traditional acute leukaemia chemotherapy only selects resistant clones [23, 24]. The analysis of *KTM2A* genomic breakpoint junction sequences has shown duplicated regions up to several hundred bases long from *KTM2A* and/or its partner gene on either derivative chromosomes, or deletions of several hundred bases. Topo-II creates 4-base staggered double-stranded breaks (DSB) in DNA, but also introduces single-stranded nicks as kinetic intermediates of DSB. The precision of the breakpoint junction sequences and the results of DNA Topo-II cleavage assays in treatment-related leukaemia suggest a mechanism in which two DNA Topo-II introduce separate single-stranded nicks in duplex DNA that are staggered by up to several hundred bases. Subsequent template-directed polymerisation of the single-stranded overhangs between the staggered nicks would then generate the sequence duplications. This leads to a DNA damage-repair model in which various naturally occurring DNA Topo-II poisons induce DNA Topo II-mediated damage in leukaemia pathogenesis [25, 26].

One interesting observation concerns the latency of t-MN and early-age leukaemia. The latency period to develop t-MN is much shorter for *KTM2A*-r than the latency period of t-MN with unbalanced aberrations, for example, monosomy-5 or monosomy-7 [27]. Important to mention that the clustered oxidative DNA lesions are a direct risk to genome stability observed in t-MN [26]. Recent studies performed using next-generation sequencing have identified additional gene mutations in *IDH1, ASXL1, SRSF2, SF3B1, SETBP1, TP53* and *KTM2A*-r in childhood t-MN [27]. These mutations were tracked backwards in bone marrow samples before the t-MN occurrence demonstrated the clonal evolution in t-MN with some somatic mutations preceding cytotoxic treatment and favouring leukemic development. Therefore, these genes are under scrutiny to test possible associations with environmental factors in the pathogenesis of i-AML.

Accepting the minimal two-step model for AML, in which the disruptive effect of abnormal gene rearrangements within cell signalling pathways originates a clone, failure in DNA repair system and/or modulation of susceptibility genetic factor, particularly with continued exposure to genotoxic substances, have been the pathogenic ripple, which needs to be uncovered.

## Overview of MLL recombinome in i-AML

*KTM2A* drives essential biological processes through its DNA bind domains either directly (through sequences enriched for AT rich or nonmethylated CpG) or indirectly (through sequence specific transcription factors such as E2Fs), providing interfaces for the assembly of multiprotein complexes and methylate histone H3 at lysine 4 [28]. Chromosomal translocations involving the human *MLL*, nowadays called *KMT2A* gene, were associated with a dismal outcome in ALL, while in AML, some reports have demonstrated that some *KMTA2* partner gene displays either a good or intermediate prognosis [3, 10, 29]. The break points for rearrangements in *KMT2A* occur mainly in 8-kb area between exon 8 and 13 (breakpoint cluster region) and accounts for 5%–10% of all acute leukaemia. In AML, *KMT2A*-r is recurrently associated with 35%–50% of infants and 5%–10% of therapy-related cases. The identification of the partner gene is necessary to provide optimal treatment stratification at diagnosis and prognosis.

German investigators with a huge worldwide collaboration have created the *MLL recombinome study* that has been allowing us to identify new reciprocal translocations, complex rearrangements, internal duplications, deletions or inversions on the 11q23 region where the *KMT2A* is located and inserted into other chromosomes [30]. According to data from *MLLrecombinome*, the most frequent rearrangements occur either with *MLLT3*/*AF9*, *MLLT1*/*ENL*, *ELL*, *MLLT10*/*AF10*, *MLLT4*/*AF6* or* AFF1/AF4* genes, or are derived from gene internal duplications (*MLL-*PTDs) representing ~90% of AML cases. Amongst infants, breakpoints occur more frequently in *KMT2A* intron 11 [31]. However, breakpoint distribution for *MLL*-*AF6* fusions displayed a clear preference for *KMT2A* intron 9 recombinations. Their occurrence differed significantly in the cohorts of infant, paediatric and adult leukaemia patients [30]. It is worthwhile to mention that the distribution of *KMT2A-r* in AML differs from ALL, either in infants, paediatric or adult patients, suggesting different biological mechanisms that may also reflect diverging factors. The extensive *MLL recombinome* analyses at the molecular level have allowed the identification of more than 71 different *KMT2A-*fusion partners [32]. To systematically classify the acute leukaemia with *KMT2A-r*, a multiplex RT-PCR (mRT-PCR) technique was standardised to identify the most frequent partners genes, such as *MLLT1*, *MLLT3*, *MLLT4*, *MLLT10*, *AFF1* and the partial tandem duplication (*MLL-*PTD) [33]. In this context, the most common *KMT2A* fusion partners in our series of i-AML are: *MLLT3/AF9* (35.7%), *MLLT1/ENL* (15.3%*)*, *MLLT4/AF6* (14.1%), *MLLT10/AF10* (10%), *AFF1/AF4* and *MLL-*PTD (6.1%). *KMT2A* with other partner genes or nonidentified are still underestimated because of the difficulty in their detection using only conventional cytogenetic technique, FISH and mRT-PCR. The *KMT2A*-r was morphologically more associated with AML-M4/M5, although was also identified in other AML subtypes, e.g., AML-M7. These groups altogether were associated with adverse prognosis [34].

The genome-wide technologies that demonstrated the genomic landscape of acute leukaemia may unravel the prenatal origin, throughout mutational signatures associated with environmental exposures.

## Childhood AML associated with maternal pesticides exposures

Consensual evidence about risk factors and etiology of c-AML is still a challenge. It is well accepted that ionizing radiation exposures during gestation and genetic syndromes, e.g., DS, and familial monosomy 7 increase the risk of AML [35]. However, those factors account for only a small proportion of cases.

It is well known that children are more susceptible to environmental carcinogens than adults due to physiological vulnerability [36]. The immaturity of tissues, higher growing rates and cellular proliferation demand might jeopardise the haemopoiesis when the child is exposed to hazard substances [36]. Regarding this, exposures to pesticides through parental occupation or household chemicals have emerged as risk factors for childhood leukaemia and, particularly, c-AML [35].

Two meta-analyses showed that prenatal maternal occupational exposure to pesticides was strongly associated with c-AML. The risk estimates were (sOR: 2.64, 95% CI: 1.48–4.71) and (mRR 2.68; 95% CI 1.06–6.78) in each study [37, 38]. Indeed, a remarkable association amongst farm-related exposures and childhood leukaemia was observed (sOR: 2.44, 95% CI: 1.53–3.89) [37]. Those results were based on few studies that assessed risk associations for c-AML individually, e.g., without grouping with ALL within analysis.

A hospital-based case-control study from Children’s Cancer Study Group (CCG), which gathered 204 AML cases younger than 18 years and single-matched controls, showed a suggestive dose-dependent association of maternal occupational exposure to pesticides with c-AML risk in the offspring (*p* for trend = 0.008). Seven out of 204 c-AML had mothers exposed to pesticides for more than 1,000 days; for either parent exposed for more than 1,000 days, the association with c-AML was significant (OR: 3.8, 95% CI: 1.5–9.7) [39]. Another CCG study tested the risk association between paternal military service and childhood leukaemia in their offspring, considering the fact that during the 1980s, militaries were exposed to toxic substances, including 2, 3, 7, 8-tetracholodibenzo-*p*-dioxin used during the Vietnam War. A total of 605 c-AML and individually matched controls were included in the analysis, and results showed that c-AML risk was slightly increased among the offspring of veterans who had served in Vietnam (OR: 1.7, 95% CI: 1.0–2.9) [40]. More recently, a pooled analysis by the Childhood Leukaemia International Consortium (CLIC) gathered data from ten case-control studies, accruing 1,357 c-AML cases and 12,443 controls; in this study, c-AML risk was increased in the offspring of mothers who were occupationally exposed to pesticides during pregnancy (pooled OR: 1.94, 95% CI: 1.19–3.18) [41]. This group has also performed a meta-analysis including CLIC studies and other data published world-wide, and found that maternal occupational exposure to pesticide during pregnancy was associated with c-AML risk in the offspring (sOR: 3.30, 95% CI: 2.15–5.06) [41].

Residential exposure to pesticides during pregnancy was also associated with increased risk to childhood leukaemia, considering unspecified pesticides (sOR: 1.54, 95% CI: 1.13–2.11), insecticides (sOR: 2.05, 95% CI: 1.80–2.32) and herbicides (sOR: 1.61, 95% CI: 1.20–2.16). Specifically, for c-AML, an increased risk association was observed with insecticides (sOR: 1.85, 95% CI: 1.29–2.64) [42]. The same results were observed by the French group (the ESCALE Study) that a national registry-based case-control study gathered 100 c-AML and 1,681 controls. Significant associations were found between c-AML risk and maternal household use of any pesticides (OR: 2.2, 95% CI: 1.4–3.3) and insecticides (OR: 2.1, 95% CI: 1.4–3.3) [43]. More recently, the CLIC group reviewed 740 c-AML and 10,847 controls to test the time-points of maternal exposures. The risk for c-AML in the offspring was increased with maternal pesticide exposure at home within 1–3 months before conception (OR: 1.49, 95% CI: 1.02–2.16) and during pregnancy (OR: 1.21, 95% CI: 1.21–1.99) [44]. Boys were more affected by c-AML than girls with maternal exposure within 1–3 months before conception (OR: 1.77, 95% CI: 1.06–2.98) and during pregnancy (OR: 1.72, 95% CI: 1.22–2.43), as well as for c-AML diagnosed until four years with exposure to pesticides during pregnancy (OR: 2.08, 95% CI: 1.44–3.02). The main types of pesticides associated with c-AML risk were insecticides (OR: 1.55, 95% CI: 1.21–2.00) and pesticides used on pets (OR: 1.51, 95% CI: 1.07–2.12) [44].

Regarding i-AML, four observational studies demonstrated associations of infant leukaemia with maternal exposure to pesticide during pregnancy, leading to the speculation of transplacental foetal exposure with DNA damage and chromosomal translocation as early pre-leukemic events [45–48]. The paucity of adequately powered studies is due to very low incidence of infant leukaemia. Only through collaborative studies is it possible to draw consistent conclusions. The first international collaborative study on infant leukaemia was conducted by Alexander *et al* that gathered patients with different costumes and genetic backgrounds (European, Middle East, South America and Asia countries) and accrued 74 i-AML cases (29 with *KMT2A*-r identified); they have shown that maternal pesticide exposure during pregnancy was associated with i-AML (OR: 5.08, 95% CI: 1.84–14.04) [45]. Then the Brazilian Collaborative Study Group of Infant Acute Leukaemia carried out a study taking into account the hypothesis of transplacental exposures with DNA damage. Sixty-two i-AML were recruited, excluding DS children, and pesticide exposures were analysed and reported since the first trimester of pregnancy until breastfeeding periods. Results have shown that maternal exposure to pesticides during pregnancy was associated with risk for overall infant leukaemia in crude analysis (OR: 2.23, 95% CI: 1.58–3.16); then, when the analysis was performed for i-AML, adjusted for sex, income, maternal age and birth weight, the risk association was (OR: 3.50 95% CI: 0.01–6.11) [46, 48].

Children exposure to pesticides occurs in different ways, from foetal period to late childhood, either through contamination of their parents’ work clothes or direct household residues in water, air, soil and food [42, 49]. Newborns are exposed across the placenta and through breastfeeding. The broad term of pesticide exposure might mislead conclusions because of the broad term, very well pointed out by Hernández and Menéndez [50]. In recent epidemiological studies, the household pesticides have been exploring separately [47, 48]. The Children’s Oncology Group within the 172 i-AML cohort showed no significant associations between household pesticide exposures, including insecticides, moth control, rodenticides, flea or tick control, herbicides, insect repellants and professional pest exterminations as variables and risk for infant leukaemia [47]. However, in Brazilian studies, maternal exposure to pesticide was associated with risk for i-AML diagnosed until 11 months of age (adjusted OR: 5.01, 95% CI: 1.97–12.7). The maternal exposure to pyrethroid (aOR: 3.39, 95% CI: 1.72–16.78) or organophosphates (OP) (aOR: 5.50, 95% CI: 1.44–21.03) was both associated with an increased risk for i-AML [48]. In a small study of infants born in an agricultural region in the Philippines, the prevalence of AML translocation with *RUNX1-RUNXT1* in cord blood samples was about 2-fold higher among those with detectable meconium levels of the methylcarbamate insecticide propoxur [51]. Another interesting study is the association found between solvents exposure and AML previously analysed in a case-study carried out among adults in Italy. An association of the disease in individuals with *RAS* mutations and antecedents of exposure to solvents was observed (OR: 4.8, 95% CI: 1.2–18.8) [52, 53]. This finding is very interesting, because *RAS* mutation genes have been observed with variable frequencies (~15%–20%) in AML [54] and *RAS* mutations in early-age leukaemia were modulated by *NQO1* rs1800566 (C609T), emphasizing the critical role of genetic susceptibility with somatic mutations in the mechanistic pathway leading to leukaemia in childhood [55].

The applications of various pesticides in agricultural and public health programs have caused severe environmental pollution and health hazards. It is a challenge to evaluate the exposure of individual substances (qualitative studies), although, Sala *et al* have measured the internal dose levels of organochlorines in paired settings of dyads, mother blood and cord blood samples from a rural village (Flix, Catalonia and Spain) located in the vicinity of an organochlorine-compound factory plant. All newborns presented detectable levels of organochlorine compounds, with higher values in samples from Flix than nearby villages [56]. This study demonstrated that the dissemination of pesticides compounds can exercise effects on nontarget organisms.

Brazil has been the world’s top pesticide market consumer as very well described by Albuquerque *et al* [57]. Therefore, epidemiological and experimental research must be performed in order to increase the level of information on the role of pesticides in i-AML.

## Speculations of possible mechanisms how pesticides compounds drive leukemogenesis

Pesticides are chemical compounds that include insecticides (organochlorines, OP, carbamates and pyrethroids), herbicides (paraquat, diquat and 2,4-dichlorophenoxyacetic acid), fungicides (dithiocarbamates and captan), fumigants (ethylene dibromide and methyl bromide), rodenticides (anticoagulants to control rodents), and algicides, among others. Since the class of organochlorine came in restricted use, the second line of pesticides, i.e., OP and pyrethroids, became the most common group available [58]. For instance, the OP insecticide chlorpyrifos (O,O-diethyl O-3,5,6-trichloro-2-pyridyl phosphorothioate) has been widely used in indoor pest control (mainly for cockroaches and termites). Some *in vitro* studies (human cell lines) have demonstrated the genotoxic potential of OP inducing DSB and hypermethylation in *CDKN1A* [59]. The genotoxity of fungicides with the concentration of dose-response to Topo-II inhibition and induction of DSB was demonstrated in fruit flies [60]. It has been demonstrated that chlorpyrifos induces *KMT2A*-r through caspase 3-pathway with genomic instability and Topo-II inhibition in human foetal liver haematopoietic CD34+ cells [61].

It has been shown that pesticides may induce oxidative stress by producing free radicals, enhancing lipid peroxidation, causing a drastic activity of antioxidant enzymes in mammalian systems [62]. Moreover, these pesticides producing oxidative stress lead to toxicity in *in vitro* experiments (animal studies), and are found also in pesticide’s manufacturing workers and sprayers [63, 64]. Thus, DNA damage and oxidative stress are proposed mechanisms that link pesticide compounds to human pathogenesis of diseases observed in epidemiological studies [65, 66]. Long-lasting or acute oxidative stress disturbs cell metabolism. The reactive oxygen species (ROS) are able to produce permanent changes in the structure of proteins, lipids and DNA. The proteins that are oxidised may lose or enhance their activity. Moreover, the proteins oxidised are able to form aggregates that inhibit the systems responsible for protein degradation and lead to alterations of proteins in the cell [67].

Pesticide structures may be very stable, which means that they do not break down easily and can remain in the environment long after application and in organisms long after exposure. Some of these compounds present with slow degradation and subsequent bioaccumulation. In summary, the biological evidence linking pesticide compounds suggest that drugs interfering with DNA remodelling by Topo-II can mediate the formation of specific chromosomal translocation breakpoints in a similar pathway as chemotherapy agents.

## Early-age AML, genetic susceptibility and genes–environment interactions

Genetic susceptibility has emerged as an important risk factor for childhood leukaemia, mainly because gene variants have modulated the attributable risk between environmental exposures and diseases. GWS have been providing lists of gene mutations, single nucleotide variations (SNP), expression frequencies and copy number variation (CNV) of candidate genes as potential biomarkers for genomic instability and novel therapeutic targets for children and adult cancer [68, 69]. However, very few studies have been conducted underlying genetic susceptibility in early-age AML with GWAS approaches.

Genetic syndromes are well established factors associated with high predisposition for childhood leukaemia and it accounts for about 10% of cases [35, 70]. A sum of multiples, independent or complementary genetic lesions are required for the development of a malignant disease from a haematopoietic progenitor clone. Maternal and infant diet, smoking, pesticides, household chemicals, automobile smoke and paint are the most important environmental risk factors for childhood leukaemia. Thus, polymorphisms in genes related to the metabolism of procarcinogenic substances may increase the risk of developing the disease. With the rationale of t-MN, the profile of genomic aberrations found in i-AML as well as the case-control studies, we performed investigations in several genes along the xenobiotic, gene variants in the base-excision repair (BER) and nonhomolog junction repair (n-HJR) systems. The enzymes of the xenobiotic system are capable of directly influencing the activation or inactivation of these compounds in the body. SNPs in gene involved with immune system pathways, cell cycle, DNA repair, folate metabolism and methylome process are also investigated in risk susceptibility to childhood leukaemia [71].

The equilibrium between the activities of Phase I and II enzymes of the xenobiotic system is essential for the organism’s response to environmental insults to haemostasis, efficiency and maintenance of human genetic material. A systematic review that included the great majority of the studies carried out in this topic has identified about 50 studies that found consistent results with the estimated rates of genetic susceptibility and risk of pediatric leukaemia. The polymorphisms most frequently investigated were those located in the genes of the cytochrome P450 family (CYPs), *GSTM1*, *GSTT1* and *NQO1* [72]. Some studies have shown that certain haplotypes of *CYP1A1* are associated with an increased risk for paediatric leukaemia (ALL and AML) whose parents are smokers (OR: 2.1) [73], while CYP2E1* 5 allele in heterozygosity was associated with the risk of AML in children in Turkey (OR: 4.9) [74]. The *GSTM1* and *GSTT1* genes encode for glutathione S-transferases μ1 and θ1, respectively, which function in detoxifying processes of electrophilic xenobiotics, such as chemical carcinogens, and environmental pollutants, and inactivate secondary metabolites from endogenous oxidative stress. *GSTM1* and *GSTT1* alleles can both be deleted (null genotypes), which result in the absence of the protein [75–77]. In our hands, the *GSTM1/T1* null genotype conferred increased risks to AML (aOR: 2.14).

The genetic variant *NQO1* 609C > T (rs1800566) known as NQO1*2 allele encodes a nonsynonymous mutation (Pro187Ser) that confers the lack of enzyme activity, and has been associated with the increased risk for benzene poisoning among benzene-exposed workers (7.6-fold rate) [78]. The *NQO1*gene encodes for a cytosolic flavoenzyme, NAD(P)H dehydrogenase quinone 1, that catalyses the two-electron reduction of quinones to hydroquinones, preventing the formation of ROS generated by redox cycling of semiquinones, thus functioning as a detoxifying enzyme [79].

The detoxification enzyme NAD(P)H: quinoneoxidoreductase (NQO1) is a flavoenzyme that detoxifies benzene metabolites, quinines and azo-dyes. NQO1 has an important function to protect cells against mutagenecity from free radicals and oxygen metabolites [79]. The *NQO1* C609T (P187S) polymorphisms have been strongly associated with the risk of childhood ALL particularly for IL with *KMT2A*-r [80]. We have found that *NQO1* C609T modified risk in pediatrics leukaemia depending on age range and the variant genotype in combinations with other gene polymorphisms, such as Paraoxonase 1 (*PON1*) gene variant [75]. PON1 functions are to oxidize and hydrolyse metabolites of several organophosphorus pesticides. Its functional activity is very important in the cellular oxidative stress process [81]. The most common *PON1* polymorphisms A21439G (PON1 Q192R) and T12801A (PON1 L55M) cause variability in enzyme activity, affecting its sensitivity to xenobiotic metabolism in young individuals [82]. We have investigated *NQO1* and *PON1* polymorphisms associated with early-age leukaemia considering acquiring genomic aberrations and age at the onset of the disease. We have found that infant leukaemia with *KTM2A*-r was strongly associated with *NQO1* C609T variant genotypes (OR: 2.93), while *PON1*L55M polymorphism increased the risk of ALL in children aged ≥13 months (OR: 3.2) [75].

Genetic polymorphisms conferred by SNPs in the DNA repair genes pathways have also been associated with genetic susceptibility to paediatric leukaemia. For instance, a Taiwanian study, which included 266 cases of paediatric leukaemia, found that XRCC4 (x-ray repair cross complementing 4) haplotypes, composed of SNPs rs6869366 and rs28360071, were associated with the increased risk for the disease, without distinguishing subtypes [83]. There is as yet no evidence in the literature regarding genetic susceptibility to AML specifically. We are performing genotyping studies with the aim to identify gene variants in BER and n-HJR systems in order to estimate the associations with i-AML/*KTM2A* positive. BER is crucial in the elimination process of single-strand damages with reinsertion of band religation. In homeostasis, the XRCC1 is responsible for recruitment and anchoring ligase-enzymes during the double-strand religation. So far, *XRCC1* 399A > G confers an increased risk for i-AML as whole. In addition, in our investigation, the DIP3-*XRCC4* was found associated with infants *KTM2A*-r (OR: 3.2). In a model system to investigate the function of XRCC genes, the DIP3 (ins/del, 30pb of intron3) of *XRCC4* had substantially decreased the XRCC4 protein levels leading to reduced cellular ligase IV activity *in vitro* and *in vivo* studies.

## Conclusion

In this review, we have revisited early-age AML, a group that comprises distinct AML with *KTM2A*-r and is associated with transplacental exposures. We have comprehensively summarised the epidemiologic and biological evidence that sustains the hypothesis of leukaemogenesis initiating upon transplacental maternal exposures. Few observational studies have estimated the risk association of i-AML with maternal exposures during pregnancy to pesticide compounds. We have put together the results of the Brazilian studies and the review of mechanistic investigations that support the biological plausibility of observational findings. The genomewide technologies that demonstrated the genomic landscape of acute leukaemia might unravel the prenatal origin throughout mutational signatures, and the challenge is to combine cell biology associated with environmental exposures. Thus, future work should explore the associations of genes involved in cell detoxification concerning the myeloid compartment and target for AML.

## Conflicts of interest

The authors declare that they have no conflicts of interest.
